# Is chronic pelvic pain a comfortable diagnosis for primary care practitioners: a qualitative study

**DOI:** 10.1186/1471-2296-11-7

**Published:** 2010-01-27

**Authors:** Linda McGowan, Diane Escott, Karen Luker, Francis Creed, Carolyn Chew-Graham

**Affiliations:** 1 School of Nursing, Midwifery and Social Work, University of Manchester, UK; 2 School of Community Based Medicine, University of Manchester, UK

## Abstract

**Background:**

Chronic pelvic pain (CPP) has a prevalence similar to asthma and chronic back pain, but little is known about how general practitioners (GPs) and practice nurses manage women with this problem. A clearer understanding of current management is necessary to develop appropriate strategies, in keeping with current health care policy, for the supported self-management of patients with long term conditions. The aim of this study was to explore GPs' and practice nurses' understanding and perspectives on the management of chronic pelvic pain.

**Methods:**

Data were collected using semi-structured interviews with a purposive sample of 21 GPs and 20 practice nurses, in three primary care trusts in the North West of England. Data were analysed using the principles of Framework analysis.

**Results:**

Analysis suggests that women who present with CPP pose a challenge to GPs and practice nurses. CPP is not necessarily recognized as a diagnostic label and making the diagnosis was achieved only by exclusion. This contrasts with the relative acceptability of labels such as irritable bowel syndrome (IBS). GPs expressed elements of therapeutic nihilism about the condition. Despite practice nurses taking on increasing responsibilities for the management of patients with long term conditions, respondents did not feel that CPP was an area that they were comfortable in managing.

**Conclusions:**

The study demonstrates an educational/training need for both GPs and practice nurses. GPs described a number of skills and clinical competencies which could be harnessed to develop a more targeted management strategy. There is potential to develop facilitated self- management for use in this patient group, given that this approach has been successful in patients with similar conditions such as IBS.

## Background

Chronic pelvic pain (CPP) is a common condition amongst women of reproductive age, but the underlying patho-physiology remains poorly understood. CPP is characterized by lower abdominal or pelvic pain that is of at least six months duration, which maybe continuous or intermittent. The condition is not specifically related to either the menstrual cycle or sexual intercourse [1]. Chronic pelvic pain is surprisingly common with approximately 38 per 1000 women attending primary care services each year with CPP, a rate comparable to that of asthma and back pain [2]. Furthermore, community surveys report between 15 and 24% of women aged 18 to 50 years old report experiencing CPP within the last three months [3,4].

Many factors have been implicated as causal, including endometriosis, pelvic inflammatory disease and adhesions [1,2]. Laparoscopy (a surgical technique which allows the pelvic organs to be visualized) is used by gynaecologists to facilitate diagnosis. However, laparoscopy fails to identify underlying pathology in up to 35% (range 3-92%) of women [5]. This means that many women do not receive a 'medical explanation' to account for their pain. This can lead to women entering a cycle of re-investigation and re-referral. The reliance on laparoscopy, as both a clinical and research tool, to assign women with CPP into known pathology versus unknown pathology categories has led to the emergence of an oversimplified, 'dualistic' model of these complex pain phenomena. Past research has tended to focus on differentiating 'organic' from 'non-organic' pain using psychological characteristics (e.g. anxiety and depression) and this has led to the emergence of a negative psychological profile for women with CPP, despite a meta-analysis producing findings to the contrary [6]. This negative profile is more commonly associated with women for whom no underlying pathology can be found to account for the pain. Thus, they suffer the same stigmatizing characteristics ascribed to other chronic illnesses, such as chronic fatigue syndrome and fibromyalgia, which are difficult to diagnose and have similar uncertain illness trajectories [7,8].

Approximately 38 per 1000 women present to primary care each year with chronic pelvic pain [2], a rate comparable to that of asthma and back pain. Despite this prevalence, little is known how this complex condition is understood and managed in primary care. Health care policy now focuses on improving the quality of care for patients with long-term conditions (e.g. NHS Improvement plan) [9] and promotes a model of care whereby patients are encouraged to self-manage their condition, alongside medical management and support. Women with CPP, particularly where no organic cause has been identified to account for their symptoms might benefit from this approach which has been used in other, possibly similar conditions such as IBS [10]. However, there is still a limited understanding about how GPs and practice nurses currently manage women with CPP in primary care.

Managing women with CPP has been reported to be frustrating for both general practitioners (GPs) and patients: this group of women has been described by GPs as 'heartsink' patients, being considered difficult to manage and treat [11]. This was most evident in cases without a definite medical explanation, where management was varied and idiosyncratic. Many women who present with CPP are reported to be dissatisfied with current management and may disengage from seeking medical care despite ongoing symptoms [12,13]. Little has been written about the role of the practice nurse with CPP women. Yet given the prevalence of CPP and the developing and important role of the practice nurse in managing patients with other long term conditions in primary care, in line with current health policy [14], it is highly likely that they will encounter this patient group.

Complex conditions, like CPP, where in a substantial number of cases the cause of the pain remains medically unexplained, provide an ongoing challenge to current management strategies in primary care. This study aimed to explore views of GPs and practices nurses on their experiences of consulting with women with CPP and understanding how they currently manage these patients.

## Methods

This qualitative study used in-depth, semi-structured face-to-face interviews with GPs and practice nurses. Ethical approval was obtained prior to the commencement of the study (number LREC 05/Q1401/60).

GPs and practice nurses were purposively sampled from publicly available practice lists in North West England. This was supplemented by the attendance of LMc at local Practice Nurse Forums, utilising a snowballing technique to recruit practice nurses. The aim was to achieve maximum variation and diversity in our sample. GPs and practice nurses were invited to participate by letter which was followed up by a phone call to ascertain their willingness to participate. 174 GPs and 33 practice nurses were approached. In total twenty-one GPs and twenty practice nurses agreed to take part in the study. Table 1 provides a summary of the characteristics of participating GPs and practice nurses.

**Table 1:** Characteristics of participating GPs (n = 21) and Practice Nurses (n = 20)

	Participating GPs n (%)	Participating PNs n (%)
Modal class age in years (range)	50-59 (30-59)	40-49 (20-59)

Male	4 (19%)	-

Female	17 (81%)	20 (100)

Practice size		
Single-handed	1 (5)	1 (5)
2-3 GPs	8 (38)	14 (70)
>3 GPs	12 (57)	5 (25)

Contractual status		
GMS	19 (90)	Not known
PMS	2 (10)	

Practice list size (range)	1,800-12,300	2,000-7,000

Known interest in		
Women's Health/Gynaecology	9 (43)	Not known

Known interest in		
Mental Health	4 (19)	Not known

Interviews with GPs and practice nurses were all carried out at their respective practices. Topic guides for GP and practice nurse interviews were developed from the existing literature, and in line with qualitative methodology this was revised and refined in response to the ongoing analysis of the interview transcripts (Table 2). Only one major change was implemented in the interview schedule, when it became apparent that practice nurses had problems recognising and talking about this condition and had limited experience of women after negative investigations. Thus, for practice nurses the interview schedule was adapted and a case scenario was inserted to elicit views about this topic. All interviews were tape recorded with written consent of the participants.

**Table 2:** Interview topics guides

GP Interview Topic Guide	Practice Nurse Interview Topic Guide: *As for GPs with additional case scenario*
• What do you understand by the term chronic pelvic pain (CPP)?	Case Scenario:
• What do you think causes women to have CPP?	*What happens if a woman has had several investigations, seen the GP on a number of occasions, been referred to a gynaecologist and has had a laparoscopy, which was negative, and then she presents to you saying: "They haven't found anything wrong, but I've still got the pain?"*
Symptoms/Pathology	
• What has been your experience of women with CPP?	
• What would be your management of women with CPP?	*Prompts*
Diagnosis/Referral/Negative Findings	• *If she wants an explanation for her symptoms where would she go?*
• What sort of intervention(s) are there in Primary Care for this patient group?	• *How would you explain her negative results to her?*
Own practice/psychological support/Information provision/Other services/Role of practice nurse/Self management	• *Would you see this woman again?*

The interviews were transcribed *verbatim*; this data was supplemented by fieldnotes. Codes were used to conceal participants' identities, anonymity and confidentiality were assured. The data was managed using the qualitative software package NVivo 7, and analysed using the principles of Framework analysis [15,16]. Thus, data were coded, extracted and then charted using the framework method. The authors (LMc, CCG and DE) read the transcripts individually and completed initial analysis, before framework and themes were agreed through discussion.

## Results

The themes from the original analysis are given in Additional file 1: Table S3. Themes presented in this paper are those of understanding CPP, making the diagnosis by exclusion, and that CPP is viewed as an intractable problem.

### Understanding chronic pelvic pain

#### A new disorder?

Several GPs suggested that CPP was a particularly difficult condition to define and classify, recognising the uncertainty of dealing with apparently unexplained symptoms. One GP raised the issue of whether this was a new problem, and questioned the use of the label 'chronic pelvic pain'.

*'I've never really thought of any condition as being chronic pelvic pain. So it's like a new description, I know all about IBS, and CPP doesn't spring to mind as a diagnosis I'd put on a computer very often. I suppose, thinking about it, since you first emailed, and I've not seen anyone since then, which is what, two weeks, who's come in with pain in the lower abdomen, related. I would imagine chronic pelvic pain is supposedly related to menstruation and women of menstrual age. So I was thinking, I don't know what the diagnosis is, even if I'd seen someone with lower abdominal pain that was recurrent, I wouldn't have thought of using that title. So it's almost like a new disease entity*.' (GP 3.)

However, GPs were more aware than practice nurses of the labels such as CPP to account for symptoms of pain which cannot be explained by organic pathology. Practice nurses, tended to focus primarily on diagnostic categories and labels. They were also uncomfortable talking about symptoms which could not be explained in bio-medical terms. Nearly all GPs could talk about at least one patient fitting with their definition of CPP. It was generally viewed as lower abdominal/pelvic pain that was variable and had been ongoing for months for which a cause had not been identified.

*'But to me it's a pain that you've tried in every way to solve, by surgery, by pain killers, by treating what you feel is the underlying condition, but that pain has not gone away*.' (GP 17.)

*'Not as chronic pelvic pain, as you say. It was dealing with chlamydia, dealing with PID, dealing with you know - ovarian cysts, polycystic ovaries but not pelvic pain... 'Cos it's like the other way about isn't it. Rather than a symptom of PID is pelvic pain rather than pelvic pain and what could it be?*' (PN 14.)

GPs readily acknowledged the possible overlap in symptoms between CPP and IBS. One GP noted that the similarity between the two conditions should be treated with caution:

*'And sometimes pelvic pain can be misdiagnosed as, you know, probably possibly irritable bowel syndrome, but you have to be careful that you don't put it down to irritable bowel syndrome as it may be something more serious gynaecologically*' (GP 6).

In contrast, practice nurses attributed their lack of awareness of CPP to their lack of training. One nurse explained that despite doing several courses related to women's health, she had not covered CPP specifically:

'*No - huge gap, bearing in mind that I trained in 1980s...I've been on quite a few and if it was an issue, it certainly should be introduced perhaps in the Cytology Module, 'cos that's an ideal opportunity, you know what I mean, if your seeing women on a regular basis, or should be, as least three yearly, so if there's any chronic ongoing thing it would be picked up at that, you know what I mean but no, definitely not'*.(PN 717.)

### Diagnosis by exclusion

Not all GPs were comfortable applying the diagnostic label of CPP to a woman, preferring only to use this term when underlying physical pathology had been excluded, or were confident that the problem was largely due to psychological phenomenon.

*'...to an extent I would see it as a diagnosis, possibly of exclusion, that perhaps might be arrived at after various sort of acute, or acute on chronic, conditions had been excluded, treated, eliminated if you like.... pain... without, an organic basis, or with no demonstrable underlying physical pathology and that's kind of my working definition of it*.' (GP 21.)

*'Well, at that stage I would be, I wouldn't be identifying it as chronic pelvic pain, I would never make as I say, I don't use the term, so I wouldn't be thinking, you know, I wouldn't be thinking that at all. I certainly wouldn't be thinking of it until I had ruled out any sort of explanation*.' (GP 16.)

Practice nurses did not feel that they had any role to play in the diagnostic process other

than delegated tasks of taking swabs from women:

'*I've not got the power of diagnosis*...'(PN 706)

*'If they said to me that they were tender or whatever I would take some swabs and suggest that they make an appointment to see the doctor when the swabs were back, because you're getting into the realms of diagnosis and I feel that's not really my role*' (PN 704.)

The lack of cohesion between GPs' and practice nurses' understanding of CPP was reflected in the adoption of different management styles, and level of involvement with this patient group. These included:

#### a) Exclude the physical

The most frequent diagnostic strategy focussed on excluding underlying physical pathology. GPs played an active role in this stage. This process appeared to be well defined for the GPs, many of whom described similar diagnostic workups and symptom management strategies. However, this process concentrated on 'excluding the physical', rather than a seeking a diagnosis that could explain the symptoms.

*'And then if you've done the investigations, ultrasound normal, bowels working fine, probably end up doing, if their periods are at all irregular I might do hormone tests, check for diabetes, try them on medications such as, uh, antispasmodics, anticholinergics, uhm...*' (GP 3.)

In contrast the majority of practice nurses described a more restricted role whereby they performed the investigative tasks delegated to them by GPs.

*'...they come to me specifically to have swabs done...*' (PN 714.)

#### b) Include the psychological

This appeared to be the preferred option for GPs once pathology had been ruled out and the women re-presented.

*'Well, depression. Stress, you know, any sort of kind of stress, stress related issues. Can always make things....I was going to say seem worse, I don't mean seem worse, 'cos they are worse, you know, they feel worse and yet if you were to, I suppose, in some way if it were possible, if you were to sort of say, well this is × amount of pain and that is × amount of pain, but you feel it as × times 2 because you're depressed. But you can't actually measure that. So one gets the impression that that's what happens*.' (GP 12.)

Whilst practice nurses showed awareness of psychosocial issues, and acknowledged that women might find CPP a difficult condition to cope with, they did not appear to directly address these issues in practice.

'Probably not (coping) very well, I think if somebody's telling you there's nothing wrong with you, and yet you're in a lot of pain, it must be absolutely, well I know it is, it is absolutely soul destroying and then you start to self-doubt yourself. Is it me?' (PN 709.)

#### c) Function of referral

GPs described referral of some women with chronic pain mainly to gynaecology, gastroenterology, pain clinics and psychological services, but respondents described a lack of availability of the latter two services and questioned the usefulness of any referral. Gynaecologists were seen as being the least useful in the management of this patient group.

*'I would always try and keep them out of gynaecology because I find that once I've referred them to gynaecology it's a gravy train, and then they, they go to gynaecology and they get pushed from this investigation to that investigation to the other investigation and basically they never get better (laughing) gynaecology never let go of them either...*' (GP 14.)

The decision to refer was felt to be driven by patient expectations, as well as their own lack of appropriate skills to manage these women:

*'I mean I don't know whether it (pressure) is necessarily coming from the patient, it might be a pressure from my own sort of inability to make people better but I, I do feel there is pressure from, from the patients. Because I do see, see that it is, I find it very difficult to try and bring patients round to looking at ways of coping with the pain, or maybe looking at psychological inputs, or a psychological reason for the pain, I find that extremely difficult*.' (GP 14.)

*'...sometimes we feel that we've done what we can, you know, what more can we do and I think that's where, you know, people with a psychological mindedness about the work that they do in primary care would still regard that as something to take on. Other people don't feel confident about it or really don't know what to do, and it may well be that, that, that the literature would also be helpful to them in that, you know, they may not be the right person to do it themselves, but they might well be able then to facilitate a person getting help elsewhere*.' (GP 8.)

Practice nurses did not view their role as referrers outside the practice, with all practice nurses stating that they would always refer a patient back to the GP:

*'Occasionally, if somebody were to, I would guess that if somebody just presented with pelvic pain, they would probably make an appointment with the GP but if they did come to see me first, I would still refer them to the GP but I would take a chlamydia swab and I would take an ordinary HVS and just sort of get a background and a history and then ask them to make an appointment with the GP when the results are back so that, in other words, that background and investigation, part of that's been done but I don't feel that I'm qualified to actually do anything more than that*.' (PN 717.)

### An intractable problem

#### Therapeutic nihilism

GPs and practice nurses acknowledged that CPP was a difficult problem to manage both for themselves, and for the women who presented to them with this condition. However, ultimately it was the GPs who managed this group of patients until either diagnosis was confirmed, the problem resolved or the women re-presented with what were considered as other medically unexplained symptoms.

*'Yeh, I think, I mean, the problem is, as always, that time's an issue and, uhm, and these patients are often people who have got named seats in the waiting room, so you see them very frequently. And when you look back over the years almost everything's been tried for them, uhm, and I mean there does come a time with some patients where you say, well I'm sorry, this is something you'll just have to live with*.' (GP 20.)

A sense of failure and frustration permeated the GP and practice nurse interviews:

*'I don't know. It's just massively frustrating, and I mean we know ourselves, because if somebody comes to see me and they've got chronic pelvic pain and they've come to see me lots of times before and they're not getting any better I feel frustrated and I feel down hearted before we start as well. And the fact that people with chronic pelvic pain do make me feel frustrated, means that people probably aren't particularly satisfied, doesn't it, I meant these are the sorts of things that, these are the sorts when you feel people aren't satisfied*.' (GP 14.)

*'Oh, nightmare! Because I'd just feel that there was nothing I'd be able to offer her because I'm not aware of anything that's there for her. It would cross my mind, which is awful, whether it is a psychological thing, which is that's awful because there's nothing worse than having this thing and nobody's taking you seriously but again, if a Consultant has failed and the GP, what role, do you understand the position I'm in? What could I offer where they have failed and as yet, I don't really know?*' (PN 717.)

#### Awareness that women disengage

GPs suspected that some women probably discontinue the route of seeking help and treatment from their GP.

*' There's probably a group who attend and attend and re-attend and frequently attend, then there's a group who feel they're not getting anywhere and they feel they're mithering their doctor so they don't attend*.' (GP 2.)

*'... I'm sure there are lots of people, I can imagine women saying that they feel dismissed...*' (GP 8.)

The reasons why were less clear, it may be that women begin to normalize the pain, or discontinue seeking medical help because they feel dissatisfied with their consultations. However, practice nurses acknowledged that women may be left feeling they have to manage the pain themselves.

*'Perhaps women think that it's just due to something to do with their menstrual problem... so it's a gynaecological thing and it's something that they've just got to put up with. And perhaps some people think that it's normal to have some of this pain. It may be too that they perhaps get very little sympathy and support from partner, husband or whatever, uhm, and so that makes them just keep on putting up with it*.' (GP 7.)

*'...the professionals are probably frustrated that you can't actually offer anything constructive in way of a diagnosis, which is often a problem with anything that's chronic that you draw on expertise that you have available, and you've explored avenues...sometimes if you draw a blank, its quite frustrating and that reflects and the patient will go away feeling that however they manage it, they've got to manage it themselves, because there doesn't seem to be a readily available solution or management strategy for them really*.' (PN 702.)

*'If they're coming back, but they are probably not coming back, the majority of them, they think - oh I'm wasting everybody's time - they think I'm neurotic. I think there is probably an awful lot of women, as I say, putting up and shutting up'*. (PN 713.)

GPs noted some women's use of alternative sources of help, through alternative therapies and media literature; especially if they are unhappy and the pain has a large impact on their life. It was acknowledged that these routes were only available to women who could afford them.

*'And I suspect, but I don't have evidence, to show that this group of patients are more likely to do that because, over a long period of time they're quite likely to become disenchanted with conventional medicine, and I'd turn to alternatives*.' (GP 20.)

*'Certainly when patients do come they've always read the sort of ladies' magazines, and I think perhaps now information is around a lot more than it used to be. Whereas for it for some people it will encourage them to seek attention, for a lot of others, particularly for something like chronic pelvic pain, where there is no easy diagnosis or easy management, easy treatment, I think a lot of women are aware of that, and perhaps don't necessarily seek treatment or help - straight away*.' (GP 6.)

The benefits of complimentary therapies were usually described by respondents in terms of extra time, relaxation, and psychological support women receive that may not be available from the NHS:

'I think it actually helps a lot of women because, partly because of the time that's spent and again it's, somebody's listening to them, as well as it's quite, it's quite a pleasant experience having the massage and the therapy, a very relaxing experience often and it's, it's a time for them.' (GP 17.)

'Uhm, and appeared to benefit from her regular sessions with the physio. Now from my point of view I'm not sure whether that was because she had an hour one to one with a person that was taking a particular interest, whereas when she comes in here we manage to see her for probably about 20 minutes because she talks the hind legs off a donkey and then, then she's gone, and certainly in secondary care you don't usually get a great deal of time with a person, so I think, uhm, there's been quite a lot of psychological support through her physio, as well as the actual physiotherapy manipulation things.' (GP 4.)

#### Clinical competence

The majority of GPs described clinical and inter-personal skills required to manage this patient group, including listening, believing, discussion, support and using reattribution.

*'Yes, we have to say that there is a pain there, and we don't know why, we believe that she has got the pain, but there is nothing seriously wrong there so she shouldn't be afraid of any cancer or any incurable disease. Also to explain to her it might not actually cause problems to her fertility in future, and the treatment option is just pain killer and maybe to do things which don't cause aggravation to her symptoms, even a small dose of antidepressant might work here and a low dose of amitriptyline as well, which is an antidepressant, but a low dose can help pain, any chronic type of pain really*.' (GP 1.)

However, most GPs suggested that their practice nurses did not possess the necessary skills or training to be able to fully engage with this patient group.

*'No, because I think managing needs is very difficult, managing people with pain syndromes is very high order skills, you need the kind of skills of, uhm, reattribution and managing somatisation, all that kind of thing, which are actually very difficult skills to acquire, and most practice nurses aren't trained to do that. I think most doctors are not very good at it either*.' (GP 15.)

*'Only in so far as doing the investigations and swabs and things. I mean we often share, particularly with one of the practice nurses, I often share care of people of all sorts, it's possible that they would get involved, but they don't have any particular training or experience to deal with pelvic pain as such...*' (GP 10.)

The majority of the practice nurses interviewed, however, suggested that they could have a more active role in delivering interventions to this group of women. This appeared to be linked with notions of easier access to practice nurses. Women may present to the practice nurse opportunistically, and the practice nurse may be perceived by women as more approachable than the GP.

*'....there is definitely a role because practice nurses are usually the first point of contact, especially when they are carrying on well woman clinics, doing cervical smears, etc new patient health checks, whatever. You know they are usually the first point of call, people will come to us, they trust us, see us as a professional person, and they can come and confide in you and get answers*.' (PN 5)

## Discussion

This study suggests that GPs and practice nurses are less comfortable making the diagnosis of chronic pelvic pain (CPP) than they would be with a more recognised, yet similar, condition such as irritable bowel syndrome (IBS). General practitioners (GPs) have reported that women who present with CPP are difficult to manage and treat, and several described this group of women as 'heartsink' patients [13]. The dichotomising of CPP into organic or non-organic aetiology has not been helpful either in understanding the presentations of or in developing treatment approaches for affected women. Grace (2000) notes that reliance on the medical paradigm has promoted the "...failure to develop understandings of the 'subjective' aspects of pain, the tendency to reduce causal processes to 'mechanisms', and the tendency to consider the psychosocial as purely reactive to the biological..." (p. 525) [17]. This dichomtomising is well described by the respondents in this study and there was agreement amongst GPs and practice nurses that women who present with chronic pelvic pain can be a difficult patient group to manage. Lack of awareness of this condition, and how it should be diagnosed and treated, offered additional threats to GPs' and practice nurses perceptions' of their own clinical competences. GP accounts reveal that a diagnosis is often reached by the process of exclusion. This process begins by excluding underlying physical pathology before the inclusion of psychosocial factors. However, if a patient continues to present with symptoms, GPs are left with limited management options, with or without the label of CPP.

Within the current organisational structure of primary care it is the GP who bears the burden of management, with practice nurses referring women back to the GP when they have reached the limit of their expertise. The GPs interviewed suggested that although they struggle with this patient group, they do not feel that the practice nurses could take over this role. Referral to secondary care was perceived to be unhelpful, and that this sometimes served to keep women in a cycle of re-referral and re-investigation until either the GP, or the woman herself, chose to disengage with this process.

Although GPs still viewed CPP as a rare and new condition, and as a difficult group of patients to manage, they were, however, empathic to the suffering of women with these symptoms. Clinical skills described by GPs in the interviews, such as listening, believing, discussion, support and using reattribution reflect those recommended for dealing with patients with somatic expression of distress or depressive symptoms [18,19]. It may be that GPs are already using these techniques in their encounters with women with CPP. Our previous work suggests this may not be the case [13]. In addition, these skills are only useful up to the point where women begin to re-present with medically unexplained symptoms and the GP may then revert to the traditional medical model. Yet, patients who present with medically unexplained symptoms have been shown to value explanation and support, rather than further investigations [20,21]. This suggests a need for training to teach those additional skills required to manage this group of patients, and prevent the cycle of unnecessary re-investigation and re-referral. Given that a large proportion of GPs caseloads consist of patients who present with somatic symptoms which GPs think are not explained by physical disease [22], acquisition of such skills would be highly valuable. The difficulty is that GPs and practice nurses will need to develop skills to help women cope with the uncertainty that symptoms of CPP brings[8] whilst dealing with the uncertainty they themselves are experiencing.

### Study limitations

This paper reports a qualitative study focussed on the management of women with CPP in primary care. A major strength was that both GPs and practice nurses were interviewed, allowing for the views of nurses on the management of CPP to be accessed for the first time. This enabled the facilitators and barriers to management of this patient group to be identified in both sets of health professionals, providing a more complete picture of the current management of CPP in primary care.

The purpose of the semi-structured interviews was to explore respondents' perceptions about CPP as a condition and the difficulties faced in managing women with CPP in primary care. The aim was not to investigate in detail previous training and education or to assess health professionals' current management strategies against guidelines, although this would be an important area to explore in future work.

The low response rate limits the generalisability of this study and those participants who were interviewed may already have an interest in women's health. The GPs and practice nurses who were interviewed did not appear to have any unusual characteristics that may suggest that were different from the population from which they were sampled (see Table 1). It was noted that after being involved in the early stages of the diagnosis, principally assisting with investigations, practice nurses appeared to be excluded from the management of women with CPP. The need for an introduction of a case scenario in the interview to help practice nurses (with limited experience of women who represent post negative investigations) engage may limit the usefulness of data describing how practice nurses might manage these women.

### Implications for practice and future research

The findings suggest that within the spectrum of chronic symptom presentation, women with CPP remain a neglected group. It was apparent from the interviews that both GPs and practice nurses would like to recognise and manage this group of women more effectively, but organisational factors within the practice and lack of clinical skills presented barriers to developing successful management strategies. Whilst organisational structures are difficult to change in the short term, clinical skills maybe more amenable to modification.

Encouraging self-management for this patient group would provide a complimentary strand to improved skills training for GPs to the overall management strategy for this patient group. The need to promote self-management of long term conditions is high on the primary care agenda [9,23] but GPs and practice nurses require the tools and skills to support the patient in coping with their symptoms [[24,25], Blakeman T, Bower P, Reeves D, Chew-Graham CA: Bringing self-management into view: a qualitative study of long-term condition management in primary care consultations, submitted], and to enable the patient to become an expert in managing their own condition [25]. Utilising the patient experience in practitioner training is vital but how this is incorporated is not yet known [26] and indeed, it has been shown that the Expert Patient Programme (EPP) might reinforce the medical paradigm [27]. Conditions with a similar profile to CPP, such as irritable bowel syndrome, have responded positively to the introduction of a self-help guide [11], the benefits of which included a reduction in consultations and perceived symptom severity at one year follow-up. A similar guide could be specifically developed for women with CPP, with the GP or practice nurse acting as the facilitator.

## Conclusions

The study demonstrates an educational/training need for GPs and practice nurses. Analysis reveals that there is a need for additional training and information for most GPs and practice nurses if they are to be better equipped to help women with pelvic pain. With such doubts about the diagnosis, the intractable nature of the condition and that GPs and practice nurses feel they have little to offer these women, combined, sometimes, with negative attitudes, there is a clear indication for further training and more specialised knowledge about CPP. A way forward would be to combine further training of practitioners about CPP with the principles of self-management. Training across the practice team to increase understanding of CPP is vital so that women no longer feel there is no help available to them [13]. This study has revealed an opportunity to develop self-help management strategies for the primary care management of CPP in women. GPs appear to consider self-management as an option only when they have exhausted their usual bio-medical approach to management. The introduction of supported self-management earlier in the process of care may help to prevent women disengaging from care and also to feel comfortable to manage their symptoms earlier in the time course of their illness. It will be important to harness the listening skills and empathy described in GPs' and practice nurse interviews, and clinical skills suggested by GP respondents, to develop a facilitated self-management intervention for these women. Such clinical skill development will help primary care professionals better manage women with CPP, as well as gaining other generic skills that are necessary to encourage patients with long term conditions to feel confident to self-manage.

## Competing interests

The authors declare that they have no competing interests.

## Authors' contributions

LMc designed and conducted the study, and drafted the manuscript. CCG, KL and FC contributed to the design and supervision of the study. LMc and DE collected data. LMc, CCG and DE analysed the data. CCG, KL, FC, DE participated in revising the manuscript. All authors have read and approved the final manuscript.

## Pre-publication history

The pre-publication history for this paper can be accessed here:

http://www.biomedcentral.com/1471-2296/11/7/prepub

## Supplementary Material

Additional file 1 **Table S3 Thematic Chart**. A chart which displays themes/subthemes from the qualitative interviews with GPs and PNs. Seven main themes were identified. Click here for file
