# Supplementary material for: Is chronic pelvic pain a comfortable diagnosis for primary care practitioners: a qualitative study
Source: BMC Fam Pract. 2010 Jan 27;11:7. doi: 10.1186/1471-2296-11-7 (PMC2835666; doi:10.1186/1471-2296-11-7)
Supplement: Additional file 1 — Table S3 Thematic Chart. A chart which displays themes/subthemes from the qualitative interviews with GPs and PNs. Seven main themes were identified. [file 1471-2296-11-7-S1.DOC]

| Table 3: Thematic Framework | | |
| --- | --- | --- |
| THEMES | SUBTHEMES | |
| General experience of gynaecological problems:  - 1. *Presentation of gynaecological*   *problems* | - Not common in relation to other priorities (e.g. diabetes; NSF sets priorities) - See a range gynaecological problems (e.g. dysmenorrhoea; PID; discharges; menorrhagia; menopausal; STDs) but PNs do not necessarily enquire about pain history - Lack of protocol/guidelines/information re: gynaecological conditions | |
| 1.2 *Understanding chronic pelvic pain* | - Both groups viewed CPP as a condition that was difficult to define, manage and treat - PNs lack awareness (not covered in training; no leaflets) – but “know it’s there” - PNs felt they saw less cases of CPP than GPs - GPs much more familiar with this condition than PNs; use the term CPP as an ‘umbrella’ diagnosis | |
| Diagnosis by exclusion 2.1 *Exclude the physical* | - Several GPs not comfortable with label of CPP until all underlying pathological reasons excluded - GPs take a detailed history and initiate investigations - PNs more task oriented – carry out GP requested investigations only (not autonomous) - Some PNs use initiative – instigate own plan; investigations; offer advice (autonomous) - PN role limited to preparing patient for consultation with GP – act as patient advocate - PNs “know their boundaries” regarding diagnosis - In some practices only GPs perform vaginal examinations | |
| 2.2 *Include the psychological* | - Most GPs will only explore psychological issues when pathology excluded - Both groups showed some awareness as to possible effects on women’s quality of life (including sexuality) - Women need to feel their pain is accepted as “real” | |
| 2.3 *Function of referral* | - Formal referral to secondary care – always instigated by GP - In some practices PN could follow-up cases and investigations – but rarely does so; system geared for GP to follow-up - Referral often not seen as helpful – particularly gynaecologists - Psychology services and Pain Clinics – not readily available so “no point” in referring women - If a woman represents to PN she is often referred directly back to GP - Other agencies – refer to surgery counsellor/mental health nurse, GU clinic, other support agencies - Some PNs – subtle re-referral back to GP – prepare patient with an agenda | |
| An intractable problem 3.1 *Therapeutic nihilism* | - Both GPs and PNs display unplanned, unsystematic, idiosyncratic strategies – unable to deal with medically unexplained cases - Several PNs had no cohesive strategies – do nothing or re-refer to GP - Both groups feel a sense of failure and frustration | |
| 3.2 *Awareness that women disengage* | - Both GPs and PNs were aware that women may disengage from seeking medical care - Women get frustrated; unhappy due to the lack of a diagnosis - Women displayed stoicism – “they just put up with it” | |
| 4. Access to practice nurses:4.1 Direct route | | - Patient self-refers PN (PN female; perceive nurses have more time; can talk more to nurses; try out things pre GP; back door to GP) - Patient attends a clinic run by PN |
| 4.2 *Indirect route* | | - Via GP – for investigations; menstrual history etc. (access varies with gender of GP; female GPs tend to deal with gynae themselves) - Women present for a smear - Women present for new patient check - Women present with “other” problem |
| 4.3 *Practice influences* | | - Practice philosophy - Receptionist as “gatekeeper” – they filter either to GP or PN - Gender of GP affects both access to PN and subsequent management - Some PNs work as triage nurses; see acute cases |
| 5. Practice nurse/GP relationship 5.1 *General issues* | | - Attitude of GP to PN role crucial - GPs directly employ PNs - Varies from practice to practice - Working in teams (link to Integrated Teams) - PNs “know their boundaries” |
| 5.2 *Role differences* | | - Gender of GP – if there is a woman GP in practice PNs see less gynaecology cases - GPs have the ‘power of diagnosis’ - PNs have more time - PNs more able to talk to patients (esp. re: sensitive issues) - Nurses perceive that they adopt a more holistic approach |
| 6. Management of gynaecological conditions (including CPP)6.1 General | | - PNs tend to be task oriented – carry out GP requested investigations only (not autonomous) - A few PNs use their initiative – instigate own plan; investigations; offer advice (autonomous) - Take a history – details specific to each practitioner - Prepare patient for consultation with GP - Act as patient advocate - Only GPs do PV examinations - Nurse practitioner can request scans (“officialised” by GP); has formal and informal access to secondary care - A few GPs had planned strategies for medically explained cases |
| 6.2 *Follow-up* | | - PN can follow-up cases and investigations – but rarely does so; system geared to GP follow-up |
| 6.3 *Referral* | | - Formal referral to secondary care – always instigated by GP - Gynaecology referrals not viewed as ‘helpful’ - PNs refer problem directly back to GP - Refer to surgery counsellor/mental health nurse - Refer GU clinic - Refer support agencies - PN subtle re-referral back to GP – prepare patient with an agenda |
| 6.4 *Medically unexplained symptoms* | | - PNs no cohesive strategies – do nothing or re-refer to GP - Both GPs and PNs reported unplanned, unsystematic, idiosyncratic strategies – unable to deal with MUS cases - Some awareness that this is a difficulty |
| *6.5 Dealing with emotional/psychological problems* | - Feel able to deal with these issues up to a point - Links in with how they see their role (autonomy; training etc) - Women mainly present spontaneously; not asked as part of routine consultation - Some women present with psychosexual problems | |
| 7. Women with chronic pelvic pain (CPP)*7.1 PN perceptions of women with CPP* | - Some awareness as to possible effects on women’s QOL (including sexuality) - Women frustrated; lack of diagnosis - Stoic – “they just put up with it” - Need to feel their pain is “real” | |
| 7.2 Other services women may use | - Family planning clinics - Counsellors - Pain clinics - A & E depts - NHS direct - Complimentary medicine | |
